# Human placenta mesenchymal stem cell-derived exosomes delay H_2_O_2_-induced aging in mouse cholangioids

**DOI:** 10.1186/s13287-021-02271-3

**Published:** 2021-03-22

**Authors:** Wenyi Chen, Jiaqi Zhu, Feiyan Lin, Yanping Xu, Bing Feng, Xudong Feng, Xinyu Sheng, Xiaowei Shi, Qiaoling Pan, Jinfeng Yang, Jiong Yu, Lanjuan Li, Hongcui Cao

**Affiliations:** 1 State Key Laboratory for the Diagnosis and Treatment of Infectious Diseases, Collaborative Innovation Center for Diagnosis and Treatment of Infectious Diseases, The First Affiliated Hospital, Zhejiang University School of Medicine, 79 Qingchun Rd., Hangzhou City, 310003 China; 2 National Clinical Research Center for Infectious Diseases, 79 Qingchun Rd., Hangzhou City, 310003 China; 3 Zhejiang Provincial Key Laboratory for Diagnosis and Treatment of Aging and Physic-chemical Injury Diseases, 79 Qingchun Rd., Hangzhou City, 310003 China

**Keywords:** Exosomes, Primary sclerosing cholangitis, Senescence, Cholangiocytes, Organoids

## Abstract

**Background:**

Cholangiocyte senescence is an important pathological process in diseases such as primary sclerosing cholangitis (PSC) and primary biliary cirrhosis (PBC). Stem cell/induced pluripotent stem cell-derived exosomes have shown anti-senescence effects in various diseases. We applied novel organoid culture technology to establish and characterize cholangiocyte organoids (cholangioids) with oxidative stress-induced senescence and then investigated whether human placenta mesenchymal stem cell (hPMSC)-derived exosomes exerted a protective effect in senescent cholangioids.

**Methods:**

We identified the growth characteristics of cholangioids by light microscopy and confocal microscopy. Exosomes were introduced concurrently with H_2_O_2_ into the cholangioids. Using immunohistochemistry and immunofluorescence staining analyses, we assessed the expression patterns of the senescence markers p16^INK4a^, p21^WAF1/Cip1^, and senescence-associated β-galactosidase (SA-β-gal) and then characterized the mRNA and protein expression levels of chemokines and senescence-associated secretory phenotype (SASP) components.

**Results:**

Well-established cholangioids expressed cholangiocyte-specific markers. Oxidative stress-induced senescence enhanced the expression of the senescence-associated proteins p16^INK4a^, p21^WAF1/Cip1^, and SA-β-gal in senescent cholangioids compared with the control group. Treatment with hPMSC-derived exosomes delayed the cholangioid aging progress and reduced the levels of SASP components (i.e., interleukin-6 and chemokine CC ligand 2).

**Conclusions:**

Senescent organoids are a potential novel model for better understanding senescence progression in cholangiocytes. hPMSC-derived exosomes exert protective effects against senescent cholangioids under oxidative stress-induced injury by delaying aging and reducing SASP components, which might have therapeutic potential for PSC or PBC.

## Background

Senescent cells usually remain metabolically active but show growth arrest in G1 phase [1, 2], such that they express the expressing cell cycle related inhibitors p21^Waf1/Cip1^ and p16^INK4a^. Cell senescence characterized by activation of the senescence-associated secretory phenotype (SASP), which activates and reinforces the inflammatory reaction of bystander cells and attracts immune cells that influence the tissue repair process [3–5]. This also facilitates the progression of chronic inflammatory diseases.

Cholangiocytes are targets of various biliary diseases [6, 7]. Continuous stimulation leads to cholangiocyte damage, which in turn leads to apoptosis or entry into a senescent state when the damage is not repaired [1]. Cellular senescence is an important pathological process in diseases such as primary sclerosing cholangitis (PSC) and primary biliary cirrhosis (PBC) [7–9]. Currently, there is no effective therapy for PSC or PBC, aside from liver transplantation [6]. Nearly half of affected patients experience one or more episodes of acute cellular rejection, and ~ 25% develop recurrent disease [10] after transplantation.

Notably, there is evidence that stem cell-derived exosomes exert a positive effect on diseases such as hepatotoxic injury [11], myocardial ischemia/reperfusion injury [12, 13], and aging-induced cardiac dysfunction [14]. Watanabe et al. and Lee et al. found that stem cell/induced pluripotent stem cell-derived exosomes significantly reduced the activity of senescence-associated β-galactosidase (SA-β-gal) and the levels of cell cycle inhibitors p21^Waf1/Cip1^, in a model of bisphosphonate-related osteonecrosis of the jaw [15] and in senescent skin fibroblasts [16], respectively. However, the protective effects of stem cell-derived exosomes against PSC and PBC remain unclear.

Traditionally, cholangiocytes cultured in two-dimensional systems show low-efficiency cell growth and subtle changes in the appearance of senescent cells. However, cells cultured in three-dimensional (3D) environments, such as Matrigel (i.e., organoids), display near-physiological cellular composition and behavior, are easier to observe, and are capable of self-organization [17]. These characteristics reduce experimental complexity compared with animal models [18–20]. Additionally, there is evidence that human PSC liver-derived cholangiocytes can develop into 3D cholangioids and exhibit senescence. Notably, oxidative stress-stimulated cholangioids that originate from normal human cholangiocytes also show senescence [21, 22]. Therefore, this study used oxidative stress (H_2_O_2_) to induce senescence in cholangioids and measured several senescence-related markers: the cell cycle arrest inhibitors p16^INK4a^ and p21^WAF1/Cip1n^ [23, 24], SA-β-gal activity [25], and pro-fibroinflammatory SASP components and chemokines [26]. Oxidative stress-stimulated organoids were then co-cultured with human placenta mesenchymal stem cell (hPMSC)-derived exosomes to clarify their protective effect.

## Materials and methods

### Cell culture

293T-HA-Rspon1-Fc and Wnt-3a cells were used as described previously to generate conditional medium of Rspon1 and Wnt-3a proteins [27]. Stably transfected 293T cells producing mouse Rspon1–Fc fusion protein were obtained as a gift from Professor Enkui Duan and Xiaohua Lei at the Chinese Academy of Sciences. Wnt3a mouse subcutaneous connective tissue cells were purchased from Bei Na Lian Chuang Biotechnology Co. Ltd. (Beijing, China). Placentae were obtained from donors at The First Affiliated Hospital, Zhejiang University School of Medicine.

### Isolation of cholangiocytes from mouse liver

Cholangiocytes were isolated from adult C57BL/6 mouse liver. After exposing the liver, blood was washed out using 1× phosphate-buffered saline (PBS) via inferior vena cava injection with a portal vein cutoff. Tissues were cut into small pieces and washed with precooled Dulbecco’s modified Eagle’s medium (DMEM) (high glucose, with GlutaMAX and pyruvate, 1% fetal bovine serum, and 1% penicillin/streptomycin), then transferred to prewarmed digestion medium (DMEM with 0.1 mg/ml DNase I, 0.125 mg/ml collagenase D, and 0.125 mg/ml Dispase II) with shaking for 1.5 h at 37 °C. During digestion, the appearance of the bile ducts was checked by light microscopy at intervals of 20–30 min. Digestion was stopped using precooled DMEM and cell pellets were collected by centrifugation at 200–300×*g*. Precooled DMEM was added to centrifuge tubes, which were then kept on ice for 30 min to collect cell pellets. The second digestion was performed with TrypLE solution and DNase I 50 μg/ml for ~ 20 min at 37 °C. Finally, pellets were washed with Advanced DMEM/F-12 (with 1% penicillin/streptomycin, 1% GlutaMAX, and 10 mM HEPES) and filtered through a 70-μm filter to obtain single cholangiocytes, as described previously [27].

### Culture and passage of cholangioids

#### Cholangiocyte seeding

The desired number of cholangiocytes were resuspended in Matrigel after isolation (10,000–20,000 cells per 50 μl Matrigel). After Matrigel had solidified via incubation at 37 °C for 15–20 min, expansion medium (Advanced DMEM/F12 plus B27 [minus vitamin A], 1 mM N-acetylcysteine, 5% [vol/vol] Rspon1-conditioned medium, 10 mM nicotinamide, 10 nM [Leu15]-gastrin I, 50 ng/ml epidermal growth factor (EGF), 100 ng/ml fibroblast growth factor (FGH) 10, and 50 ng/ml hepatocyte growth factor (HGF) was added, as previously described [27]. For the first 3 days after isolation, expansion medium was supplemented with 25 ng/ml human Noggin, 30% (vol/vol) Wnt3a-conditioned medium, and 10 μM Rho kinase (ROCK) inhibitor (Y-27632). During culture, medium was refreshed at most every 2–3 days.

#### Cholangioid passage

At 7–14 days after seeding, precooled Advanced DMEM/F-12 (with 1% penicillin/streptomycin, 1% GlutaMAX, and 10 mM HEPES) was added and then pipetted up and down to disrupt the Matrigel. Organoids were collected, digested into single cells by incubation with TrypLE solution at 37 °C, and reseeded into new Matrigel. Organoids were usually passaged with a split ratio of 1:3–4 at intervals of 5–7 days after the first passage. Organoids at passages 1–4 were used in the study. Detailed reagent information is shown in Table S1.

### In vitro modeling of cholangioid senescence and administration of exosomes

After stable growth of cholangioids (2–3 days), expansion medium containing H_2_O_2_ (50 nM) was added to organoids in the senescent (Sen) group, while organoids cultured in expansion medium containing both H_2_O_2_ (50 nM) and exosomes (0.1 μg/ml) represented the exosome-treated (Exo) group. Organoids in the control group (Ctrl) were grown in expansion medium alone. All medium was replaced at intervals of 24 or 48 h for up to 120 h.

### SA-β-gal activity assessment

For complete collection of organoids, the expansion medium was aspirated and replaced with 500–1000 μl precooled Cell Recovery Solution (Dow Corning, Corning, NY, USA) per well. The plates were shaken gently at 4 °C to disrupt the Matrigel. The supernatant was carefully removed and precooled 1× PBS was used to wash the cells three times. SA-β-gal activity was detected using the senescence detection kit (BioVision, Mountain View, CA, USA). The percentage of SA-β-gal-positive organoids was determined for each condition using a 20× objective and bright field illumination of 30 randomly selected areas. In this study, 30% was set as the cutoff value: if one organoid contained more than 30% green-stained cells, it was considered SA-β-gal staining-positive.

### Immunofluorescence and immunohistochemical analyses

Organoids were collected carefully as described in the SA-β-gal activity subsection above, then fixed in 4% (wt/vol) paraformaldehyde at 4 °C and subjected to immunofluorescence or immunohistochemistry analysis.

#### Immunofluorescence

Fixed organoids were washed in cold PBS–Tween (0.1% vol/vol) solution and blocked in cold OWB (1 ml Triton X-100 and 2 g bovine serum albumin in 1 L 1× PBS). Organoids were transferred to a 24-well plate. Primary antibodies were added (anti-CK7, anti-Ki67, anti-CK19, and anti-p21^WAF1/Cip1^) and incubated with the organoids overnight at 4 °C. After the organoids had been washed in 1× PBS, secondary antibodies were added and organoids were incubated overnight at 4 °C. Organoids were removed to fructose–glycerol clearing solution (60% vol/vol glycerol and 2.5 M fructose) and incubated at room temperature for 20 min, then transferred to slides with coverslips and subjected to imaging as described previously [28].

#### Immunohistochemistry

Organoids were washed with 1× PBS and resuspended in 70% ethanol, then dehydrated and stained in 0.5% eosin (dissolved in 96% ethanol) for 30 min. The organoids were dehydrated in 100% ethanol and embedded in paraffin; the following processes were the same as the normal immunohistochemistry steps. Detailed antibody information is shown in Suppl. Table 1.

### Preparation of hPMSC-derived exosomes

Exosomes were collected from hPMSCs at passages 3–5. After hPMSCs reached 80–90% confluence, they were washed once with PBS and covered with serum-free DMEM (low glucose), then cultured for another 48 h. Subsequently, the supernatant was collected and centrifuged for 30 min at 10,000×*g* to remove cell debris, filtered through a 0.22-μm filter, and concentrated using a 100-kDa molecular weight cutoff ultrafiltration tube (Merck Millipore, Hessen, Germany). Ultracentrifugation was performed at 120,000×*g* at 4 °C for 70 min using preparative ultracentrifuges (Beckman Coulter, Brea, CA, USA). Small extracellular vesicles (exosomes) were obtained and washed once in 1× PBS, then stored at − 80 °C until use. Exosome concentrations were determined using a Micro BCA Protein Assay Kit (Thermo Fisher Scientific, Waltham, MA, USA).

### Culture medium cytokine analysis

Organoid supernatant was collected and analyzed using a LEGENDplex™ Mouse Proinflammatory Chemokine Detection Kit (BioLegend, San Diego, CA, USA) for interleukin (IL)-6, CCL2, CXCL1, CXCL10, and tumor necrosis factor (TNF)-α and a Mouse Chemokine C-X-C-Motif Ligand ELISA Kit (Elabscience, Wuhan, China) for CXCL2, CX3CL1, CXCL16, and IL-8/CXCL15. The kits were used in accordance with the manufacturers’ instructions.

### Organoid viability assay

Before organoid treatment with H_2_O_2_ and exosomes, or when organoids had reached the desired culture duration for analysis, expansion medium was replaced with working fluids, which contained 2 μM calcein and 8 μM propidium iodide, in accordance with the instructions of the Live & Dead Viability/Cytotoxicity Assay Kit for Animal Cells (Keygen Biotech, Nanjing, China). Organoids were observed and imaged using a confocal laser scanning microscopy (Zeiss LSM710; Carl Zeiss AG, Jena, Germany).

### Software packages

Images were cropped using Adobe Photoshop CS6 (version 13.0; Adobe Systems Software Ireland Ltd., Dublin, Ireland) and formatted using Adobe Illustrator 2020 (version 24.0.1; Adobe Systems Software Ireland Ltd.). Flow cytometry data were analyzed by FlowJo V10 (BD Biosciences, Franklin Lakes, NJ, USA). Immunohistochemical staining results were analyzed by Image-Pro Plus 6.0 (Media Cybernetics, Rockville, MD, USA). The number of organoids was counted by Image J (https://imagej.net/Download).

### Statistical analysis

Statistical analyses of the p16^INK4a^, p21^WAF1/Cip1^, and Ki67 immunohistochemical staining results, and the percentage of SA-*β*-gal-positive organoids, were performed with two-tailed Student’s *t* test; other date was performed with one-way or two-way analysis of variance (ANOVA), and the Sidak multiple comparisons test was used to confirm the results of the ANOVA. Data analysis was conducted using GraphPad Prism 8.0.1 (GraphPad Software Inc., La Jolla, CA, USA). *P* < 0.05 was considered to indicate statistical significance.

### Additional methods

Western blotting procedures, transmission electron microscopy (TEM) assessment of exosomes, RNA isolation and real-time quantitative reverse transcription polymerase chain reaction (qRT-PCR) procedures, and hPMSC culture are described in the supplementary material.

## Results

### Establishment and characteristics of cholangioids from mouse liver ducts

**Fig. 1:**
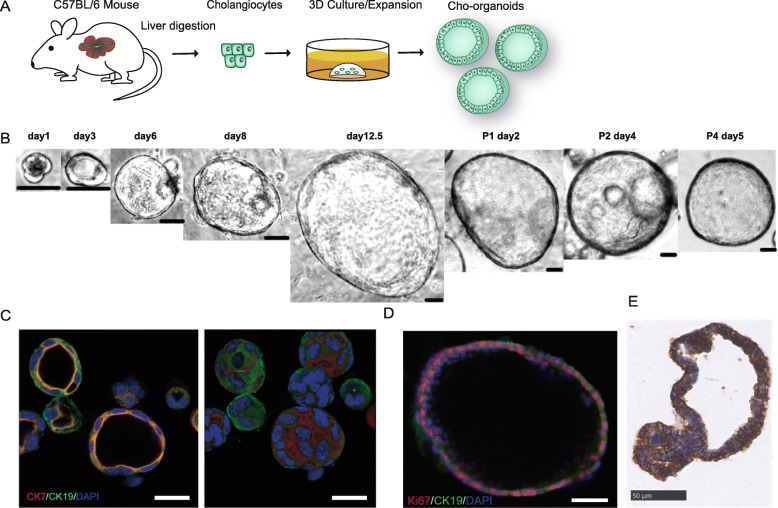
Murine cholangiocyte organoids originating from mature cholangiocytes. **a** Schematic of cholangioid development. Primary cholangiocytes collected from wild-type C57BL/6 mice by liver duct cell digestion were embedded in Matrigel and covered with expansion medium, allowing them to grow into organoids. **b** Representative image of long-term cultured cholangioids (P1, passage 1; P2, passage 2; P4, passage 4) (scale bar, 50 μm). **c** Confocal images of cholangioids expressing specific cholangiocyte markers CK19 (green) and CK7 (red), with blue DAPI staining (scale bar, 20 μm). Left: organoids in two-dimensional form; right: organoids in 3D form. **d** CK19-positive (green) cholangioids expressing proliferating protein Ki67 (red) (scale bar, 50 μm). **e** Representative immunohistochemistry images of epithelial cell adhesion molecule (EpCAM) in cholangioids (scale bar, 50 μm)

We isolated primary cholangiocytes from the livers of adult C57BL/6 mice (body weight 20–28 g) by collagenase digestion, gravitational settling, and multistep centrifugation. The cells were embedded in Matrigel and covered with conditional medium, which contained epidermal growth factor (EGF), fibroblast growth factor (FGF)10, hepatocyte growth factor (HGF), [Leu15]-gastrin I, and Wnt agonists (e.g., R-spondin 1) (Fig. 1a). Under these culture conditions, cholangiocytes grew into spheroid shapes during the first 3 days, then exhibited more rapid growth over the following 2 weeks (Fig. 1b). Immunofluorescence and immunohistochemical analysis showed that 3D spheroid organoids (Fig. 1c, right) originated from bile duct cells and expressed the cholangiocyte-specific markers cytokeratin CK7 and CK19 (Fig. 1c, left). They also expressed epithelial cell adhesion molecule (EpCAM) (Fig. 1e), a marker that distinguishes cholangiocytes from hepatocytes. Finally, proliferation protein Ki67 staining analysis revealed high proliferative activity (Fig. 1d).

### Features of long-term cultured cholangioids

**Fig. 2:**
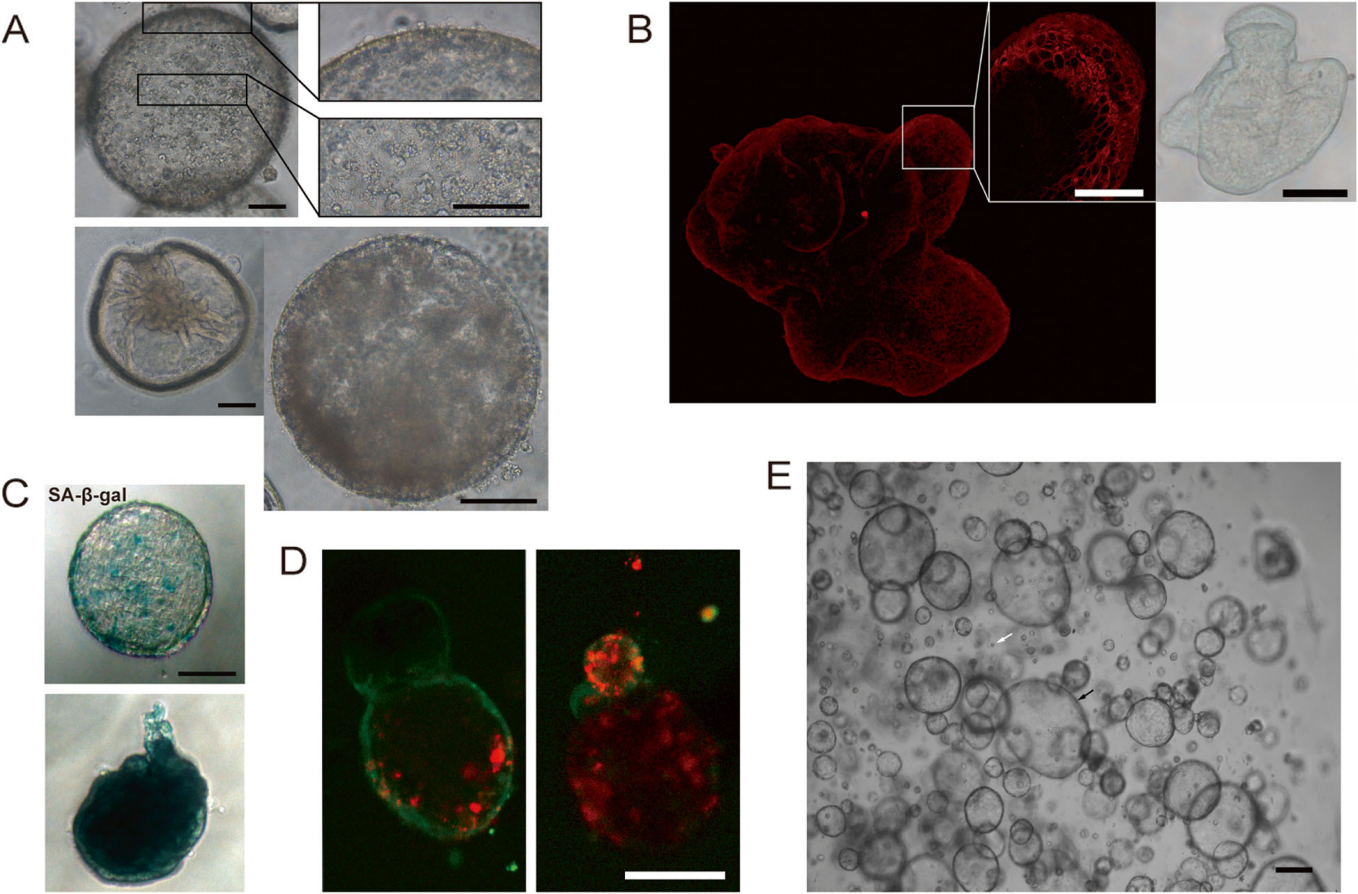
Features of long-term cultured cholangioids. **a** Organoids (P1 day 12) observed by light microscopy. Cells scattered inside and outside the organoid lumen (top, right bottom); shrunken organoids (left bottom) (scale bar, 100 μm). **b** CK7-positive cholangiocytes within organoids (P0 day 11). Cells remained close together and developed into more than one layer, as observed by confocal laser scanning microscopy (scale bar: left, 50 μm; right, 100 μm). **c** SA-β-gal staining of cholangioids (P1 day 12), some senescent cholangiocytes (green, top) and completely senescent cholangioids (bottom) (scale bar, 100 μm). **d** Viability/cytotoxicity assay of organoids (P1 day 4). Dead cells (red) aggregated inside the organoid lumen (green) (scale bar, 50 μm). **e** Organoids (P1 day 2) observed by light microscopy. White arrow indicates single cholangiocyte, and black arrow indicates organoids grown from single cholangiocytes (scale bar, 200 μm)

After 10–14 days of culture, the cholangiocytes in organoids remained in close contact and developed into more than one cell layers (Fig. 2b). Some organoids shrank in a manner like deflation of a balloon (Fig. 2a, left bottom). Other organoids had cells scattered inside and outside their lumen (Fig. 2a, top and right bottom), which were confirmed to be dead cells by viability/cytotoxicity analysis (Fig. 2d, red). Furthermore, long-term cultured organoids expressed SA-*β*-gal (Fig. 2c). After digestion into single cells and subsequent passaging, some single cells developed into organoids (black arrow), while others did not (white arrow) (Fig. 2e). Therefore, it is suggestable to passage organoids before the appearance of cell aggregates inside the lumen to increase the organoid formation rate from single cells. In our study, the plating efficiency of the different passages varied from 36.5 to 41.8% (data not shown), consistent with previous findings [27, 29].

### Persistent treatment of H_2_O_2_ induced senescence in cholangioids

**Fig. 3:**
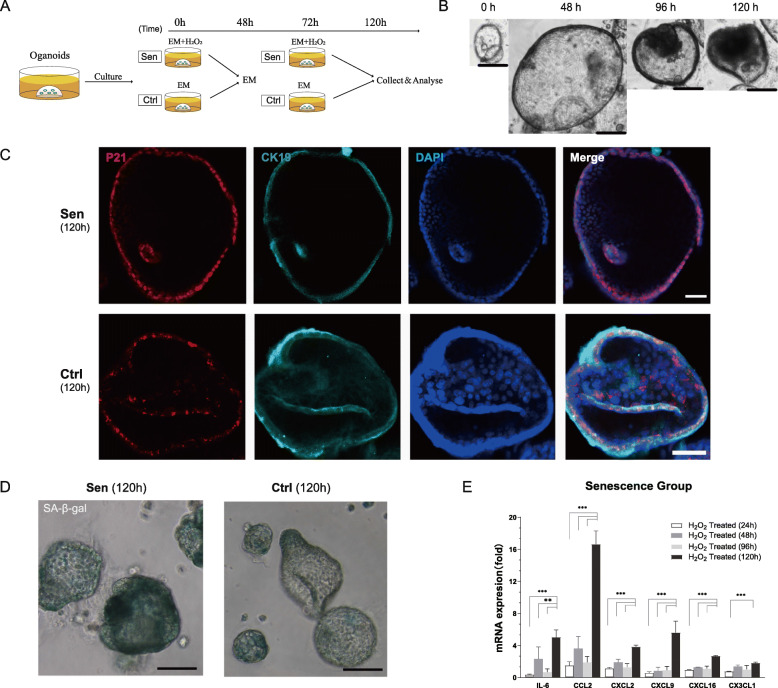
Establishment of senescent cholangioids by H_2_O_2_ treatment. **a** Schematic of the treatment of expansion medium (EM) containing 50 nM H_2_O_2_ was classified into the Sen group. The Ctrl group was treated with EM only. Medium was changed at intervals of 24 or 48 h. **b** Representative light microscopy image of H_2_O_2_-stimulated organoids, showing cytological features characteristic of senescence (reduced size, darkened spheroids) (scale bar, 200 μm). **c** Typical organoids in the Sen and Ctrl groups expressing cell cycle arrest protein p21^WAF1/Cip1^ (red) after exposure to oxidative stress for 120 h; CK19 protein was stained cyan and cell nuclei were stained blue (scale bar, 50 μm). **d** SA-β-gal-positive organoids (green) in the Sen and Ctrl groups following oxidative stress induction for 120 h, observed by light microscopy (scale bar, 100 μm). **e** mRNA expression levels of SASP components and chemokines (*IL-6*, *CCL2*, *CXCL2*, *CXCL16*, and *CX3CL1*) increased significantly at 120 h, compared with 24, 48, and 96 h. Data are presented as mean ± standard deviation (SD) (***P < 0.001, **P < 0.01, *n* = 4; ordinary one-way ANOVA)

After establishment of the organoid culture system, we tested whether exposure to oxidative stress could induce cell senescence. Before treatment with H_2_O_2_, organoid viability was verified through viability/cytotoxicity assay in which live cells were stained green (Fig. S1a). Then, 50 nM H_2_O_2_ with expansion medium was added to organoids and cultured for 120 h, as shown in Fig. 3a. The diameter of H_2_O_2_-induced organoids increased rapidly during the first 48 h, consistent with the presentation of normal organoids. After 48 h, the appearance of some organoids was like that of long-term cultured organoids (Fig. 2a): shrunken, scattered dead cells, and aggregated cells inside and outside the lumen (Figs. 3b and S1b). H_2_O_2_-treated organoids partially expressed SA-β-gal (Fig. 3d), as well as the cell cycle arrest markers p21^WAF1/Cip1^ (Fig. 3c) and p16^INK4a^ (Fig. 5b, top row, Senescence).

SASP components and some chemokines, another feature of cell senescence, were detected by qRT-PCR, and included *IL-6*, *IL-8* (Fig. S4b), *CCL2*, *CXCL2*, *CX3CL1*, *CXCL9*, and *CXCL16* (Fig. 3e). Detection of SASP components at different time points indicated that the mRNA expression levels of these components were significantly higher at 120 h than at 24, 48, or 96 h. Compared with the control group, the mRNA expression of *CCL2* increased 16.65-fold with 120 h, and 1.110–3.626-fold with 24–96 h, exposure to H_2_O_2_ treatment (*P* < 0.05). These results suggested that cell senescence was successfully induced by H_2_O_2_ treatment and that 120-h culture was adequate for establishment of senescent cholangioids. These cholangioids could be used for further research, including as a 3D model for PSC in vitro.

### Exosomes were derived from hPMSCs and co-cultured with organoids

Stem cell/induced pluripotent stem cell-derived exosomes have been shown to play protective roles in senescence-related diseases [15, 16]. Therefore, we hypothesized that mesenchymal stem cell (MSC)-derived exosomes may play a similar role in PSC.

**Fig. 4:**
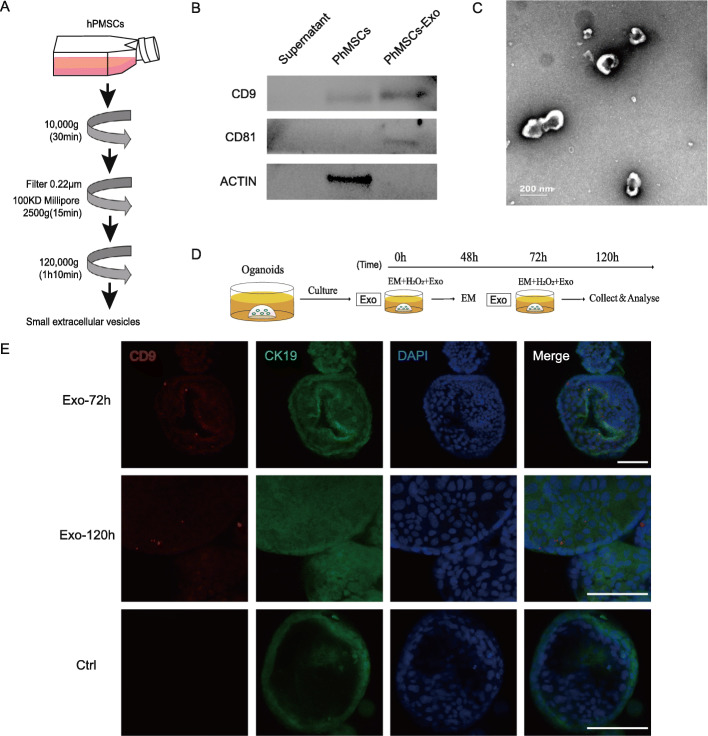
Exosomes were derived from hPMSCs and co-cultured with organoids. **a** Schematic representation of exosome collection. **b** Western blotting: hPMSC-derived exosomes expressing exosome-specific markers CD9 and CD81. **c** hPMSC-derived exosomes observed by transmission electron microscopy (scale bar, 200 nm). **d** Schematic of exosome treatment. Organoids treated with expansion medium (EM) containing 50 nM H_2_O_2_ and 0.1 μg/ml exosomes were classified into the Exo group. Cholangioids were collected and analyzed at 48, 72, and 120 h. **e** Presence of the exosome-specific marker CD9 (red) in cholangioids (CK19, green) of the Exo group after culture for 72 and 120 h, demonstrated by immunofluorescence analysis (scale bar, 100 μm)

The MSCs used in our research were derived from human placenta. They were identified through imaging (Fig. S3a), assessment of multilineage differentiation potential, and detection of MSC-specific markers. hPMSC pluripotency was verified by differentiation into osteoblasts (Fig. S3b) and adipocytes (Fig. S3c). The MSC-specific markers CD73, CD90, and CD105 were verified by flow cytometry analysis (Fig. S3d). Small extracellular vesicles were then obtained using the serial ultracentrifugation protocol shown in Fig. 4a. Western blotting revealed that MSC-derived vesicles expressed the exosome-specific markers CD9 and CD81 (Fig. 4b). Morphology analysis by transmission electron microscopy (TEM) (Fig. 4c) indicated that the small extracellular vesicles were exosomes.

We co-cultured 0.1 μg/ml exosomes with 50 nM H_2_O_2_-stimulated cholangioids to verify the protective effect reported previously [11]. Details are shown in Fig. 4d. Exosomes were confirmed to be absorbed into cholangioids after culturing for 72 and 120 h, based on immunofluorescence staining (Fig. 4e).

### hPMSC-derived exosomes delay aging in senescent cholangioids

**Fig. 5:**
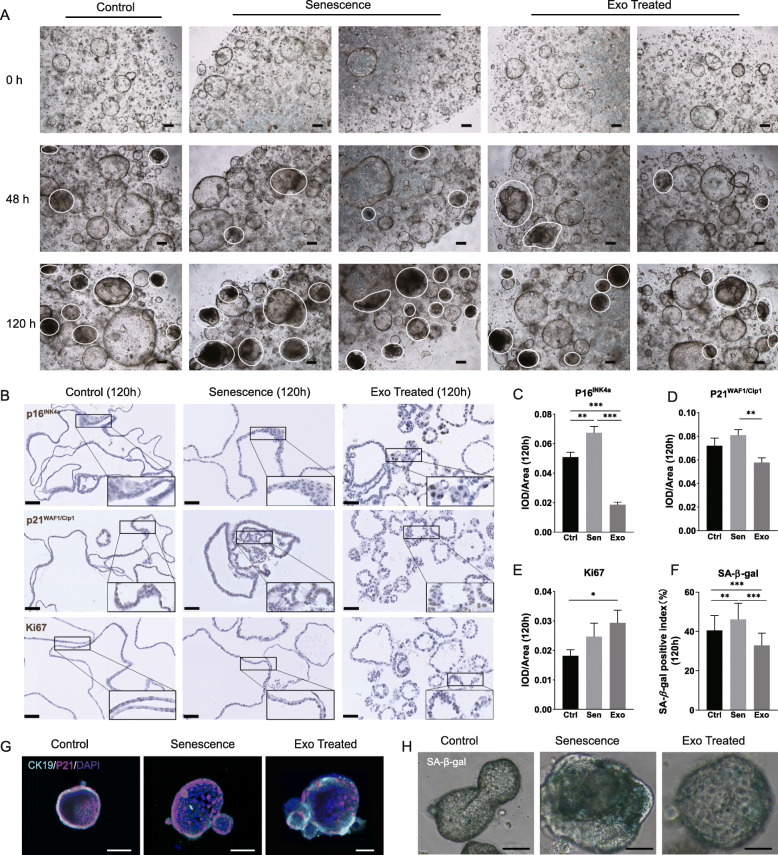
hPMSC-derived exosomes have a protective effect in senescent cholangioids. **a** Comparison of organoids among the Ctrl, Sen, and Exo groups at 0 h (top), 48 h (middle), and 120 h (bottom). Typical senescent organoids at 120 h are indicated by white circles (scale bar, 200 μm). **b** Comparison of the immunohistochemical staining results for proliferation marker Ki67, as well as the senescence-associated markers p16^INK4a^ and p21^WAF1/Cip1^ among the Ctrl, Sen, and Exo groups at 120 h (× 40 objective, scale bar, 200 μm; rectangle indicates × 80 objective). **c**–**e** Semi-quantitative analysis of p16^INK4a^, p21^WAF1/Cip1^, and Ki67 immunohistochemical staining results at 120 h. These data were acquired with a × 20 objective, and Image-Pro Plus software was used to analyze 10 randomly selected areas (two-tailed *t* test, mean ± standard error of the mean, *n* = 10, ***P < 0.001, **P < 0.01, *P < 0.05). **f** Percentages of SA-β-gal-positive organoids in 30 randomly selected areas, counted under a × 20 objective with bright field illumination (two-tailed *t* test, mean ± SD, *n* = 30, ***P < 0.001, **P < 0.01). **g** Representative CK19-positive cholangioids (cyan) in 3D form. The area of p21^WAF1/Cip1^-positive staining was greater in the Sen group than in the other two groups, while the Exo group showed the smallest area (scale bar, 100 μm). **h** SA-β-gal-positive cholangioids. Organoids were collected and analyzed at 120 h (green, scale bar, 50 μm)

After oxidative stress-induced injury and exosome treatment, we recorded changes in organoid growth from 0 to 120 h via light microscopy. We found that the number of senescent cholangioids increased gradually over time. As shown in Figs. 5a and S2a, senescent cholangioids at 120 h noted by white lines suggested that the number of senescent organoids was relatively lower in the Exo group than in the Sen and Ctrl groups. Further analysis showed significant differences in the number of senescent organoids between the Sen and Exo groups at 120 h (*p* = 0.0262) (Fig. S2b), suggesting that exosomes may delay organoid aging during exposure to oxidative stress.

To investigate this possibility, we examined the expression patterns of SA-β-gal and cell cycle arrest proteins in the Sen, Exo, and Ctrl groups. We found that the expression level of the cell cycle arrest protein p16^INK4a^ (Fig. 5b, top row) was reduced significantly by exosome treatment relative to the Sen and Ctrl groups (Fig. 5c). Exosome-treated organoids showed lower expression of p21^WAF1/Cip1^ protein than the Sen group (Fig. 5b, middle row, and 5d). This was confirmed by immunofluorescent staining (Figs. 5g and S2c). However, semi-quantitative comparison of p21^WAF1/Cip1^ expression between the Ctrl and Sen groups did not show a significant difference. Furthermore, organoids in the Exo group exhibited greater proliferative activity compared with the Ctrl group (Fig. 5b, bottom row, and 5e). These results demonstrated the ability of exosome treatment to relieve senescence and promote proliferation. Similarly, the percentage of SA-*β*-gal-positive organoids decreased significantly (Fig. 5f) (*P* < 0.05), as typical SA-β-gal-positive cholangioids shown in Fig. 5h. Interestingly, when the organoids from Sen, Exo, and Ctrl groups were digested and cultured for 1 day and 5 days, the number of second-generation organoids in Sen group was significantly lower than that in Exo and Ctrl group (Fig. S2d-2e).

Taken together, these results supported our hypothesis that hPMSC-derived exosomes exert a protective effect against senescence in cholangioids.

### hPMSC-derived exosomes reduced expression of SASP components

**Fig. 6:**
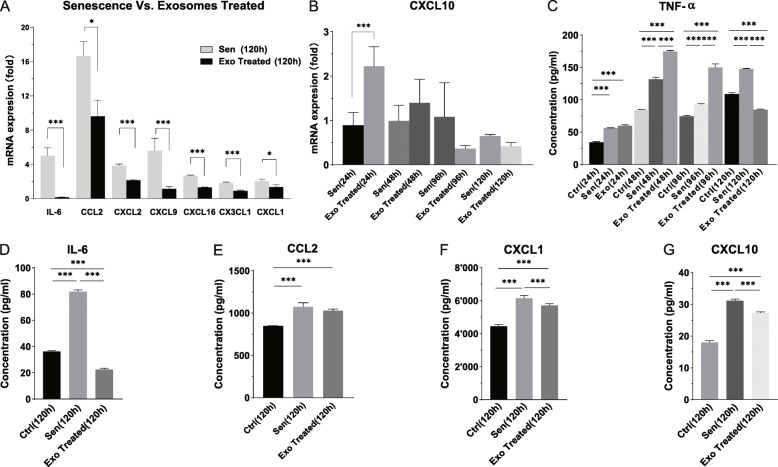
Exosomes reduced the expression of SASP components and chemokines in senescent cholangioids. **a** mRNA expression levels of SASP components (*IL-6*, *CCL2*, *CXCL2*, *CXCL9*, *CXCL16*, *CX3CL1*, and *CXCL1*), which decreased significantly at 120 h in the Exo group compared with the Sen group. Data are presented as mean ± SD (*n* = 4). **b** Fold changes of CXCL10 mRNA expression in organoids at different time points. Data are presented as mean ± SD (*n* = 4). **c** Tumor necrosis factor-α protein concentrations in culture supernatant at 24, 48, 96, and 120 h. Data are presented as mean ± SD (*n* = 3). **d**–**g** IL-6, CCL2, CXCL1, and CXCL10 protein concentrations in culture supernatant at 120 h, which were significantly decreased in the Exo group compared with the Ctrl and Sen groups. Data are presented as mean ± SD (*n* = 3). One-way ANOVA, ***P < 0.001, **P < 0.01, *P < 0.05

Our previous studies showed significantly increased expression of SASP components in senescent cholangioids. Thus, we also assessed whether exosomes could affect SASP components in this study. We evaluated the mRNA expression levels of SASP components and chemokines in the Exo group, including *IL-6*, *CCL2*, *CXCL9*, *CXCL16*, *CXCL10*, and *CX3CL1* (Fig. 6a, b), as well as *IL-8* (Fig. S4b). All tested cytokines showed an initial peak at 48 h, followed by a reduction at 96 h and a higher peak at 120 h (Fig. S4a) (all *P* < 0.001), in accordance with the trends in the Sen group (Fig. 3e). The reduction might have been caused by exchanging expansion medium at 48 h. Compared with the Ctrl group, significant reductions in mRNA expression levels were observed in the Exo group for *IL-6* (0.1518- and 5.022-fold of Ctrl, respectively) and *CCL2* (9.622- and 16.65-fold of Ctrl, respectively) (*P < 0.05, ***P < 0.001) (Fig. 6a).

We also detected the protein concentrations of SASP components by enzyme-linked immunosorbent assay and mouse proinflammatory chemokine detection kit. In the Exo group, we found that the TNF-α protein concentration also increased during the first 96 h, but was lower at 120 h, compared with the Sen and Ctrl groups (Fig. 6c). In addition, IL-6, CCL2, CXCL1, and CXCL10 protein concentrations showed decreasing trends at 120 h (Fig. 6d–g), like the changes in their gene expression levels. Notably, IL-6, CXCL1, and CXCL10 were reduced significantly compared with the other groups (all *P* < 0.001). However, the protein expression levels of CX3CL1, CXCL2, CXCL15/IL-8, and CXCL16 in the Exo group were not reduced significantly compared with the other groups (Fig. S4c–f).

Overall, our results suggested a role for SASP in the pathogenesis of senescence, and demonstrate the ability of hPMSC-derived exosomes to reduce the expression of SASP components.

## Discussion

In this study, we established organoids from mouse liver duct cells and characterized them morphologically and biochemically as 3D spheroidal structures with high expression of cholangiocyte markers. We also identified the characteristics of long-term cultured organoids, which exhibited an aged state. Persistent oxidative stress can induce senescence and, ultimately, SASP in cholangioids. Treatment with hPMSC-derived exosomes can delay the aging process, possibly through the regulation of the cell cycle-associated inhibitor proteins p21^WAF1/Cip1^ and p16^INK4a^, thereby influencing cell cycle progression. hPMSC-derived exosomes positively affected the expression levels of SASP components and chemokines in senescent cholangioids. These results are important for establishing an in vitro PSC model, and for related pathogenesis research, as well as for the development of novel therapeutic approaches.

The widely used organoid culture system was first described by Broutier et al. and Huch et al. [27, 30]. In our study, we simplified the collection of liver duct cells by gravitational settling and multistep centrifugation of digestion supernatant, rather than manual collection or flow cytometry sorting. Through the establishment of cholangioids, the growth process and morphological differences were easier to observe under light microscopy. Important, organoid culture systems enable studies of human development and disease, which are not easily modeled in animals [20, 31, 32].

Senescent cells exhibit growth arrest in the G1 phase and fail to enter the next stage of the cell cycle, which limits proliferation [3, 21]. However, in our study, organoids with oxidative stress-induced senescence were able to grow. Staining for SA-β-gal and 3D confocal microscopy for detection of p21^WAF1/Cip1^ protein did not reveal these markers in all organoid cells, which suggested that senescence was not uniform [22]. Therefore, we established a standard for easier identification and quantification of SA-β-gal-positive organoids. There is also evidence that senescent cholangiocytes can induce senescence in “bystander cholangiocytes” via paracrine signaling [33]. Thus, we suggest that cholangiocyte senescence was induced by H_2_O_2_ treatment, which led to senescence in bystander cells that eventually spread to the entire organoid. However, hPMSC-derived exosomes may reduce the spread of senescence. The digestion of senescent organoids and the number of “secondary organoids” can also show the effect of hPMSC-derived exosomes on senescence.

Immunohistochemical staining showed significant morphological differences among the three groups, which might had been caused by multiple factors. In the Ctrl group, organoids were larger due to their more rapid growth and then became deformed under the physical pressure associated with paraffin embedding. In the Sen group, in addition to physical pressure, deformed organoids may also have been caused by senescence-related shrinkage. However, in the Exo group, because of the anti-senescence effect of exosome treatment, cell growth was slowed and organoids remained in a proliferative state, without shrinkage. Therefore, most organoids exhibited a round shape in the Exo group. Furthermore, the expression of p21^WAF1/Cip1^ was not significantly upregulated after oxidative stress-induced injury in the Sen group compared with the Ctrl group, suggesting that p16^INK4a^ is more suitable than p21^WAF1/Cip1^ as a marker of aging.

MSCs/stem cells have been widely used to cure diseases. Recent studies have demonstrated the beneficial effects of MSC/stem cell-derived exosomes on senescence [14, 16, 34, 35]. To the best of our knowledge, few studies have investigated the effect of MSC-derived exosomes on cholangiocyte senescence in PSC, such that the detailed mechanism remains unknown. Some studies have demonstrated a protective effect of stem cell-derived exosomes on liver injury mice [11, 36]. Our study provides a novel 3D cell model complementing in vivo experiments showing the protective effect of hPMSC-derived exosomes. An important limitation of this study was that it did not include in vivo analysis of the protective effect of exosomes, which would be included in future experiments.

Treatment with CCL2 and CXC3L1, components of SASP, has been shown to induce cellular senescence in biliary epithelial cells, suggesting a self-amplifying secretory network in which SASP reinforces cellular senescence [26, 37, 38]. In our study, hPMSC-derived exosomes reduced the expression of SASP components and chemokines, such as CCL2, CX3CL1, IL-6, TNF-α, CXCL1, and CXCL10, which are involved in the recruitment of immune cells, terminal differentiation of B cells, and the direct damage caused by cytotoxic T lymphocytes [3]. It is plausible that exosomes exert a protective effect by reducing certain SASP components and may weaken the self-amplifying effect in senescence, thereby influencing immune cell systems and regulating the senescent microenvironment [39]. The underlying mechanisms of these putative effects remain unclear.

In conclusion, our in vitro model of oxidative stress-induced senescence in cholangiocytes can be used to study PSC and PBC. It may also be useful for investigating the mechanisms underlying the protective effect of MSC-derived exosomes in senescent cholangioids. The insights provided by this study may lead to a novel model of the mechanisms of action of senescent cholangiocytes, which could in turn lead to treatments for diseases such as PSC and PBC.

## Supplementary Information

**Additional file 1 Supplementary Material**.

**Additional file 2: Table S1.** Regents of organoids analysis.

**Additional file 3: Table S2.** Primer sequences used in this study.

**Additional file 4: Figure S1.** Characteristics of organoids in H_2_O_2_-induced senescence. (a) Viability / cytotoxicity assay of organoids before oxidative stress induction. Almost all the organoids were alive (green), with a small number of single cells dead (red) (scale bar, 100 μm). (b) Typical appearance of organoids after 120 h H_2_O_2_ induction, observed by light microscopy. Cells scattered and aggregated inside the lumen of organoids (scale bar, 200 μm).

**Additional file 5: Figure S2.** The protective effect of hPMSCs-derived exosomes on senescent cholangioids. (a) Supplement fields to Fig. 5a, organoids in Sen and Exo group at 120 h. Typical senescent organoids were noted with white lines circle (scale bar, 200 μm). (b) Analysis of randomly selective fields of senescent organoids in Sen and Exo group at 120 h (two tailed *t*-test, mean ± SD, *n* = 4, *P < 0.05). (c) Detailed immunofluorescent staining pictures of typical organoids in Ctrl, Sen, and Exo group after oxidative stress induction for 120 h. Cell-cycle-arrest protein p21^WAF1/Cip1^ were stained red, CK19 protein were stained cyan, and the cell nuclei were stained blue (scale bar, 100 μm). (d) Typical photos of “secondary organoids” passage from Ctrl, Sen and Exo group, which were cultured for 1 and 5 days (scale bar, 100 μm). (e) Numbers of “secondary organoids”. Organoids in Ctrl, Sen and Exo group were passaged at 120 h. The experiments were repeated for three times. After 1 and 5 days of culture, three fields of each experiment were randomly selected under 4 × object lens and counted using Image J software (ordinary one-way ANOVA, mean ± SD, *n* = 9, ***P < 0.001, **P < 0.01).

**Additional file 6: Figure S3.** hPMSCs culture and identification. (a) Picture of hPMSCs captured by light microscope: classic spindle-shaped morphology (scar bar, 100 μm); (b) Alizarin red S staining of hPMSCs on day 21(scar bar, 100 μm); (c) Oil red O staining of hPMSCs on day 28 (scar bar, 100 μm). (d) Flow cytometry analysis of surface antigens on hPMSCs (CD73, CD90, CD105, CD11b, CD19, CD34, CD45 and HLA-DR).

**Additional file 7: Figure S4.** Concentration of SASP components and chemokines of organoids culture supernatant. (a) Line graph, mRNA expression fold changes of SASP components at 24, 48, 96 and 120 h in Exo group (two-way ANOVA, mean, *n* = 4). (b) The fold changes of IL-8 mRNA expression of organoids at 24, 48, 96 and 120 h (ordinary one-way ANOVA, mean ± SD, *n* = 4); (c) CX3CL1 protein concentration in culture supernatant at 96 h, and 120 h. (d) CXCL2 protein concentration in culture supernatant at 96 h, and 120 h. (e) CXCL15 protein concentration in culture supernatant at 120 h. (f) CXCL16 protein concentration in culture supernatant at 120 h. Data are presented as mean ± SD (ordinary one-way ANOVA, *n* = 3).

## Data Availability

The data that support the findings of this study are available from the corresponding author upon reasonable request. I confirm that I have included a citation for available data in my references section, unless my article type is exempt.

## Abbreviations

PSC: Primary sclerosing cholangitis
PBC: Primary biliary cirrhosis
hPMSC: Human placenta mesenchymal stem cell
SA-β-gal: Senescence-associated β-galactosidase
SASP: Senescence-associated secretory phenotype
3D: Three-dimensional
PBS: Phosphate-buffered saline
DMEM: Dulbecco’s modified Eagle’s medium
EGF: Epidermal growth factor
FGH: Fibroblast growth factor
HGF: hepatocyte growth factor
ROCK: Rho kinase
EM: Expansion medium
Sen: Senescent group
Exo: Exosome-treated group
Ctrl: Control group
IL-6: Interleukin 6
TNF: Tumor necrosis factor
ANOVA: Analysis of variance
TEM: Transmission electron microscopy
qRT-PCR: Real-time quantitative reverse transcription polymerase chain reaction
EpCAM: Epithelial cell adhesion molecule
