# Supplementary material for: Human placenta mesenchymal stem cell-derived exosomes delay H2O2-induced aging in mouse cholangioids
Source: Stem Cell Res Ther. 2021 Mar 22;12:201. doi: 10.1186/s13287-021-02271-3 (PMC7983269; doi:10.1186/s13287-021-02271-3)
Supplement: Supplementary file 1 — Additional file 1 Supplementary Material. [file 13287_2021_2271_MOESM1_ESM.docx]

**Supplementary Material**

**Experimental procedures**

**RNA isolation and real-time quantitative reverse transcription polymerase chain reaction (qRT-PCR) measurement of short-term retention**

Organoids were collected as described in Cholangioid passage. To extract total RNA, culture medium was removed completely and washed in 1× PBS, 350 μl buffer RLT (RNeasy Mini Kit; Qiagen, Hilden, Germany) was added per well, and medium was vortexed vigorously in 1.5-ml Eppendorf tubes for 30 s. Reverse transcription was performed with 0.5–1 μg RNA using QuantiTect Reverse Transcription Kit (Qiagen). Real-time qRT-PCR was performed in a volume of 20 μl using the ABI 7500 Real-Time PCR System and SYBR Premix Ex Taq™ II Kit (Takara Bio Inc., Shiga, Japan). The primer sequences are shown in Table S2.

**Western blotting and transmission electron microscopy of exosomes**

Approximately 2–10 μg of protein was run on 4–15% Mini-PROTEAN® TGX™ Gels (Bio-Rad Laboratories, Inc., Hercules, CA, USA) by SDS-PAGE in a Mini-PROTEAN® Tetra Vertical Electrophoresis Cell (Bio-Rad Laboratories, Inc.) and then transferred to polyvinylidene difluoride membranes (Merck KGaA, Darmstadt, Germany). After incubation in blocking buffer (Beyotime Biotech Co., Ltd., Shanghai, China) at room temperature for 1 h, the membranes were incubated with primary antibodies to CD63 (Abcam, Cambridge, UK, diluted 1:500) and CD81 (Abcam, diluted 1:500) overnight at 4°C, then washed three times in Tris-buffered saline with 0.1% Tween (TBST; Sangon Biotech) and incubated with secondary antibodies, horseradish-peroxidase-conjugated goat anti-rabbit IgG (Abcam, diluted 1:4000). After washing in TBST, the membranes were incubated with the Pierce™ ECL Western Blotting Substrate (Thermo Fisher Scientific) for 5 min and detected with the ChemiScope Western Blot Imaging System (Clinx Science Instruments Co., Ltd., Shanghai, China).

Transmission electron microscopy analysis of exosomes was performed at the Center of Cryo-Electron Microscopy, Zhejiang University.

**hPMSC culture and characterization**

hPMSCs were isolated as previously described[29] and cultured in human MSC culture medium (STEMCELL Technologies Inc., Vancouver, Canada) in culture flasks. Flow cytometry analysis: to identify the human MSC surface markers, MSCs were harvested and incubated with PE anti-human CD34 (343505, Biolegend, San Diego, CA, USA), PE anti-human CD45 (304007, Biolegend), PE anti-human CD73 (ecto-5'-nucleotidase) (344003, Biolegend), PE anti-human CD90 (Thy1, 328109, Biolegend), PE anti-CD105 (Endoglin, 800503, Biolegend), PE anti-human CD11b (301305, Biolegend), PE anti-human HLA-DR (307605, Biolegend) and corresponding isotype controls (Biolegend) for 30 min at 4°C in the dark. The MSCs were washed twice with PBS containing 0.5% BSA (Sangon Biotech Corp., Shanghai, China), and analyzed by BD LSR II flow cytometer (Becton Dickinson Co., San Jose, CA, USA), as described previously [30].

**Pluripotency identification of hPMSCs:** cells were cultured in audiogenic and osteogenic medium (OriCell™ hMSC Adipogenic and Osteogenic Differentiation Medium; Cyagen Biosciences, Guangzhou, China), as previously described[31]. After culture for 4 weeks, Oil Red O staining (Sinopharm Chemical Reagent Co. Ltd., Shanghai, China) and Alizarin Red S staining (Genmed Scientifics Inc., Shanghai, China) were performed.
