# Supplementary material for: Human placenta mesenchymal stem cell-derived exosomes delay H2O2-induced aging in mouse cholangioids
Source: Stem Cell Res Ther. 2021 Mar 22;12:201. doi: 10.1186/s13287-021-02271-3 (PMC7983269; doi:10.1186/s13287-021-02271-3)
Supplement: Supplementary file 2 — Additional file 2: Table S1. Regents of organoids analysis. [file 13287_2021_2271_MOESM2_ESM.docx]

**Table S1. Regents of organoids analysis.**

| Antibodies | |
| --- | --- |
| Cholangioids characteristics identification | |
| Goat Anti-Rabbit IgG H&L (HRP) | Abcam, ab6721 |
| Rabbit Anti-Mouse IgG H&L (HRP) | Abcam, ab6728 |
| Recombinant Anti-Cytokeratin 7 antibody [EPR17078] | Abcam, ab181598 |
| Anti-Ki67 antibody [SP6] | Abcam, ab16667 |
| Anti-Cytokeratin 19 antibody [A53-B] | Abcam, ab194399 |
| Recombinant Anti-p21antibody [EPR18021] | Abcam, ab188224 |
| Anti-CDKN2A/p16INK4a antibody [2D9A12] | Abcam, ab54210 |
| Goat Anti-Rabbit IgG H&L (Alexa Fluor® 647) | Abcam, ab150083 |
| Goat anti-Mouse IgG (H+L) Cross-Adsorbed Secondary Antibody, Alexa Fluor 488 | Invitrogen, A-11001 |
| DAPI | BD Pharmingen, 564907 |
