# Supplementary material for: Human placenta mesenchymal stem cell-derived exosomes delay H2O2-induced aging in mouse cholangioids
Source: Stem Cell Res Ther. 2021 Mar 22;12:201. doi: 10.1186/s13287-021-02271-3 (PMC7983269; doi:10.1186/s13287-021-02271-3)
Supplement: Supplementary file 3 — Additional file 3: Table S2. Primer sequences used in this study. [file 13287_2021_2271_MOESM3_ESM.docx]

**Table S2. Primer sequences used in this study.**

| Target gene | Forward | Reverse |
| --- | --- | --- |
| CCL2/MCP-1/JE | gtggggcgttaactgcat | caggtccctgtcatgcttct |
| CCL3/MIP-1α | cccagccaggtgtcattttcc | gcattcagttccaggtcagtg |
| CCL4/MIP-1 β | ttctgtgctccagggttctc | gaggaggcctctcctgaagt |
| CCL5/RANTES | gtgcccacgtcaaggagtat | gggaagcgtatacagggtca |
| CXCLI/GROα | gcacccaaaccgaagtcata | tggggacaccttttagcatc |
| CXCL2/GROβ | cgcccagacagaagtcatag | tcctcctttccaggtcagtta |
| CXCL9/MIG | caaaatttcatcacgccctt | tctccagcttggtgaggtct |
| CXCL10/IP-10 | cccacgtgttgagatcattg | gctctctgctgtccatccat |
| CXCL16 | agctccgcagaagccggaga | agcgacactgccctggttgc |
| CX3CL1 | tcggactttgttggttcctc | caaaatggcacagacattgg |
| β-actin | ccaccgatccacacagagta | ggctcctagcaccatgaaga |
| IL-6 | tagtccttcctaccccaatttcc | ttggtccttagccactccttc |
| IL-8/CXCL15 | tcgagaccatttactgcaacag | cattgccggtggaaattcctt |
