# Supplementary material for: Human placenta mesenchymal stem cell-derived exosomes delay H2O2-induced aging in mouse cholangioids
Source: Stem Cell Res Ther. 2021 Mar 22;12:201. doi: 10.1186/s13287-021-02271-3 (PMC7983269; doi:10.1186/s13287-021-02271-3)
Supplement: Supplementary file 4 — Additional file 4: Figure S1. Characteristics of organoids in H2O2-induced senescence. (a) Viability / cytotoxicity assay of organoids before oxidative stress induction. Almost all the organoids were alive (green), with a small number of single cells dead (red) (scale bar, 100 μm). (b) Typical appearance of organoids after 120 h H2O2 induction, observed by light microscopy. Cells scattered and aggregated inside the lumen of organoids (scale bar, 200 μm). [file 13287_2021_2271_MOESM4_ESM.docx]

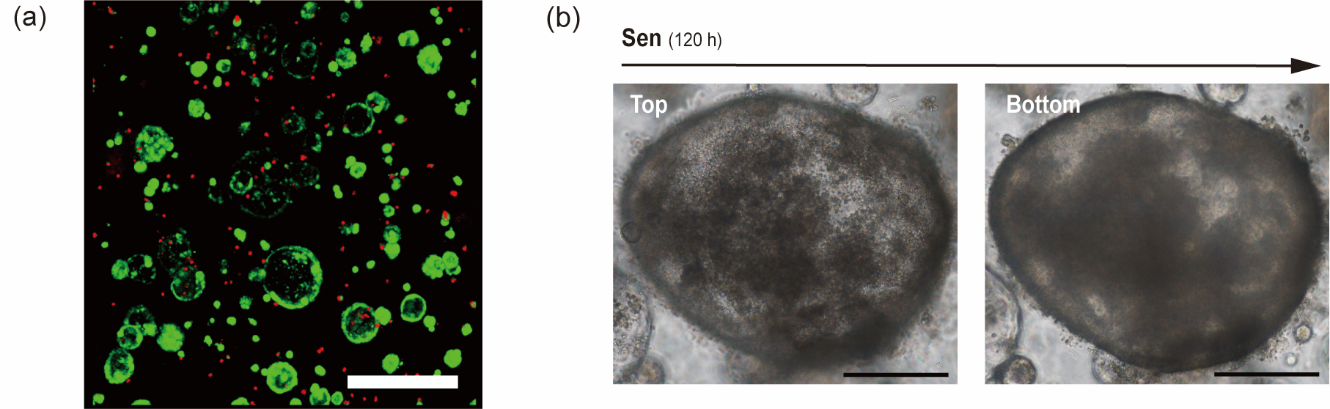


**Fig. S1** **Characteristics of organoids in H_2_O_2_-induced senescence.**

(a) Viability / cytotoxicity assay of organoids before oxidative stress induction. Almost all the organoids were alive (green), with a small number of single cells dead (red) (scale bar, 100 μm). (b) Typical appearance of organoids after 120 h H_2_O_2_ induction, observed by light microscopy. Cells scattered and aggregated inside the lumen of organoids (scale bar, 200 μm).
