# Supplementary material for: Human placenta mesenchymal stem cell-derived exosomes delay H2O2-induced aging in mouse cholangioids
Source: Stem Cell Res Ther. 2021 Mar 22;12:201. doi: 10.1186/s13287-021-02271-3 (PMC7983269; doi:10.1186/s13287-021-02271-3)
Supplement: Supplementary file 5 — Additional file 5: Figure S2. The protective effect of hPMSCs-derived exosomes on senescent cholangioids. (a) Supplement fields to Fig. 5a, organoids in Sen and Exo group at 120 h. Typical senescent organoids were noted with white lines circle (scale bar, 200 μm). (b) Analysis of randomly selective fields of senescent organoids in Sen and Exo group at 120 h (two tailed t-test, mean ± SD, n = 4, *P < 0.05). (c) Detailed immunofluorescent staining pictures of typical organoids in Ctrl, Sen, and Exo group after oxidative stress induction for 120 h. Cell-cycle-arrest protein p21WAF1/Cip1 were stained red, CK19 protein were stained cyan, and the cell nuclei were stained blue (scale bar, 100 μm). (d) Typical photos of “secondary organoids” passage from Ctrl, Sen and Exo group, which were cultured for 1 and 5 days (scale bar, 100 μm). (e) Numbers of “secondary organoids”. Organoids in Ctrl, Sen and Exo group were passaged at 120 h. The experiments were repeated for three times. After 1 and 5 days of culture, three fields of each experiment were randomly selected under 4 × object lens and counted using Image J software (ordinary one-way ANOVA, mean ± SD, n = 9, ***P < 0.001, **P < 0.01). [file 13287_2021_2271_MOESM5_ESM.docx]

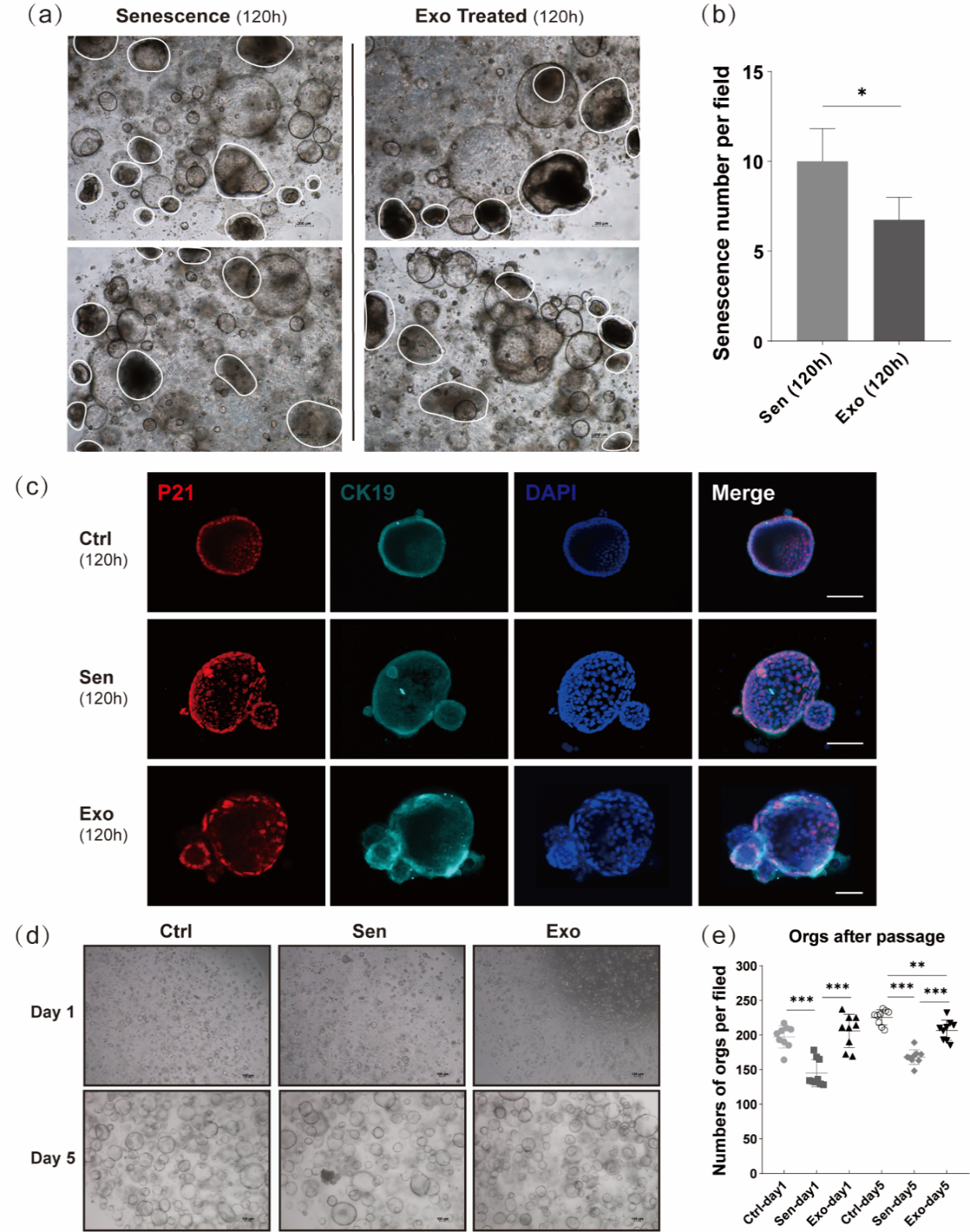


**Fig. S2 The protective effect of hPMSCs-derived exosomes on senescent cholangioids.**

(a) Supplement fields to Fig.5a, organoids in Sen and Exo group at 120 h. Typical senescent organoids were noted with white lines circle (scale bar, 200 μm). (b) Analysis of randomly selective fields of senescent organoids in Sen and Exo group at 120 h (two tailed *t*-test, mean ± SD, n=4, **P*<0.05). (c) Detailed immunofluorescent staining pictures of typical organoids in Ctrl, Sen, and Exo group after oxidative stress induction for 120 h. Cell-cycle-arrest protein p21^WAF1/Cip1^ were stained red, CK19 protein were stained cyan, and the cell nuclei were stained blue (scale bar, 100 μm). (d) Typical photos of “secondary organoids” passage from Ctrl, Sen and Exo group, which were cultured for 1 and 5 days (scale bar, 100 μm). (e) Numbers of "secondary organoids". Organoids in Ctrl, Sen and Exo group were passaged at 120 h. The experiments were repeated for three times. After 1 and 5 days of culture, three fields of each experiment were randomly selected under 4 × object lens and counted using Image J software (ordinary one-way ANOVA, mean ± SD, n=9, ***P < 0.001, **P < 0.01).
