# Supplementary material for: Human placenta mesenchymal stem cell-derived exosomes delay H2O2-induced aging in mouse cholangioids
Source: Stem Cell Res Ther. 2021 Mar 22;12:201. doi: 10.1186/s13287-021-02271-3 (PMC7983269; doi:10.1186/s13287-021-02271-3)
Supplement: Supplementary file 6 — Additional file 6: Figure S3. hPMSCs culture and identification. (a) Picture of hPMSCs captured by light microscope: classic spindle-shaped morphology (scar bar, 100 μm); (b) Alizarin red S staining of hPMSCs on day 21(scar bar, 100 μm); (c) Oil red O staining of hPMSCs on day 28 (scar bar, 100 μm). (d) Flow cytometry analysis of surface antigens on hPMSCs (CD73, CD90, CD105, CD11b, CD19, CD34, CD45 and HLA-DR). [file 13287_2021_2271_MOESM6_ESM.docx]

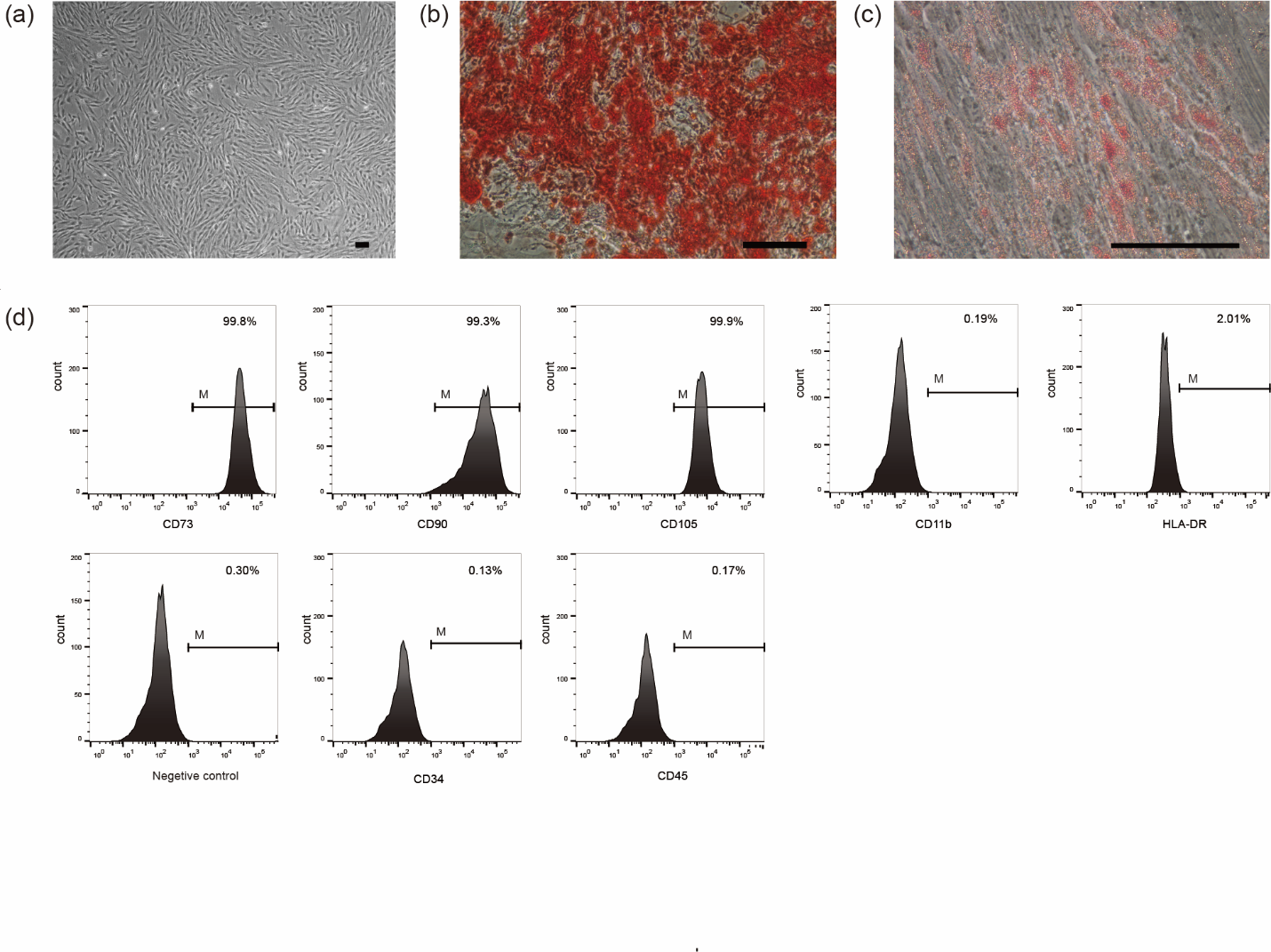


**Fig. S3** **hPMSCs culture and identification.**

(a) Picture of hPMSCs captured by light microscope: classic spindle-shaped morphology (scar bar, 100 μm); (b)Alizarin red S staining of hPMSCs on day 21(scar bar, 100 μm); (c) Oil red O staining of hPMSCs on day 28 (scar bar, 100 μm). (d) Flow cytometry analysis of surface antigens on hPMSCs (CD73, CD90, CD105, CD11b, CD19, CD34, CD45 and HLA-DR).
