# Supplementary material for: Human placenta mesenchymal stem cell-derived exosomes delay H2O2-induced aging in mouse cholangioids
Source: Stem Cell Res Ther. 2021 Mar 22;12:201. doi: 10.1186/s13287-021-02271-3 (PMC7983269; doi:10.1186/s13287-021-02271-3)
Supplement: Supplementary file 7 — Additional file 7: Figure S4. Concentration of SASP components and chemokines of organoids culture supernatant. (a) Line graph, mRNA expression fold changes of SASP components at 24, 48, 96 and 120 h in Exo group (two-way ANOVA, mean, n = 4). (b) The fold changes of IL-8 mRNA expression of organoids at 24, 48, 96 and 120 h (ordinary one-way ANOVA, mean ± SD, n = 4); (c) CX3CL1 protein concentration in culture supernatant at 96 h, and 120 h. (d) CXCL2 protein concentration in culture supernatant at 96 h, and 120 h. (e) CXCL15 protein concentration in culture supernatant at 120 h. (f) CXCL16 protein concentration in culture supernatant at 120 h. Data are presented as mean ± SD (ordinary one-way ANOVA, n = 3). [file 13287_2021_2271_MOESM7_ESM.docx]

**
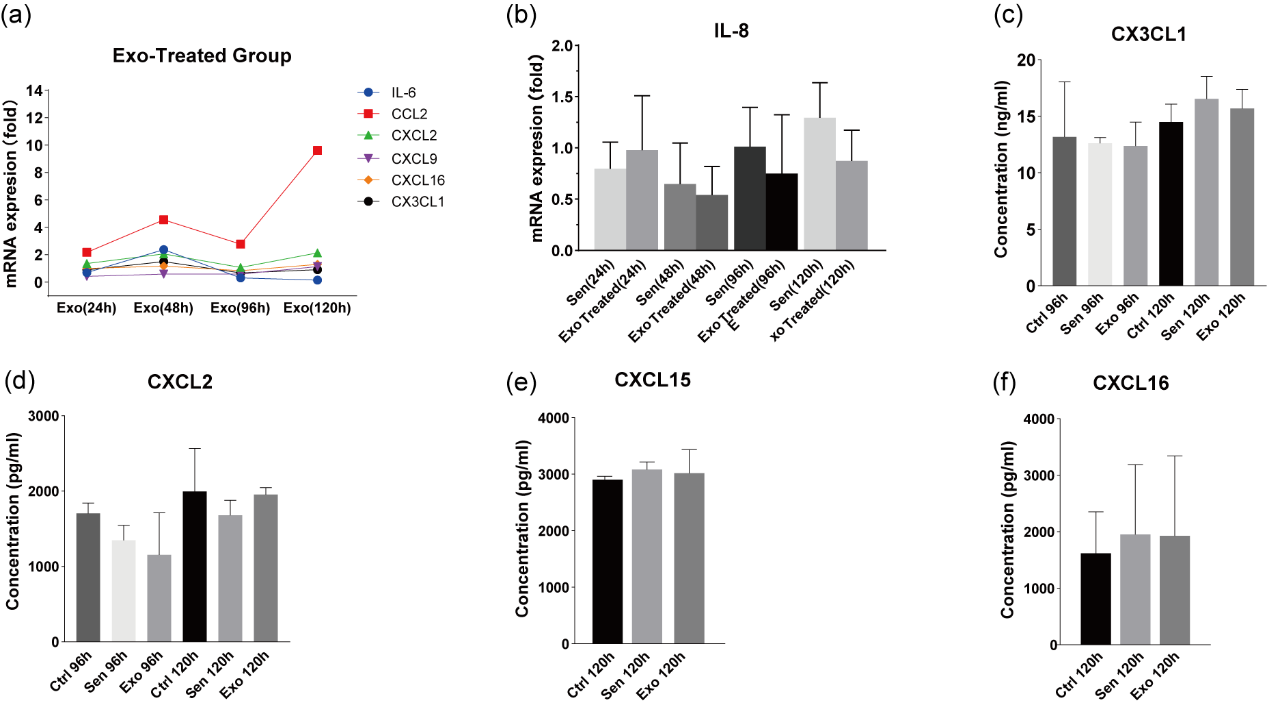
**

**Fig. S4** **Concentration of SASP components and chemokines of organoids culture supernatant.**

(a) Line graph, mRNA expression fold changes of SASP components at 24, 48, 96 and 120 h in Exo group (two-way ANOVA, mean, n=4). (b) The fold changes of IL-8 mRNA expression of organoids at 24, 48, 96 and 120 h (ordinary one-way ANOVA, mean ± SD, n=4); (c) CX3CL1 protein concentration in culture supernatant at 96 h, and 120 h. (d) CXCL2 protein concentration in culture supernatant at 96 h, and 120 h. (e) CXCL15 protein concentration in culture supernatant at 120 h. (f) CXCL16 protein concentration in culture supernatant at 120 h. Data are presented as mean ± SD (ordinary one-way ANOVA, n=3).
